# Iron deficiency, elevated erythropoietin, fibroblast growth factor 23, and mortality in the general population of the Netherlands: A cohort study

**DOI:** 10.1371/journal.pmed.1002818

**Published:** 2019-06-06

**Authors:** Michele F. Eisenga, Maarten A. De Jong, Peter Van der Meer, David E. Leaf, Gerwin Huls, Ilja M. Nolte, Carlo A. J. M. Gaillard, Stephan J. L. Bakker, Martin H. De Borst

**Affiliations:** 1 Division of Nephrology, Department of Internal Medicine, University of Groningen, University Medical Center Groningen, Groningen, the Netherlands; 2 Department of Cardiology, University of Groningen, University Medical Center Groningen, Groningen, the Netherlands; 3 Division of Renal Medicine, Brigham and Women’s Hospital, Harvard Medical School, Boston, Massachusetts, United States of America; 4 Division of Hematology, Department of Internal Medicine, University of Groningen, University Medical Center Groningen, Groningen, the Netherlands; 5 Department of Epidemiology, University of Groningen, University Medical Center Groningen, Groningen, the Netherlands; 6 Department of Internal Medicine and Dermatology, University of Utrecht, University Medical Center Utrecht, Utrecht, the Netherlands; Royal Derby Hospital, UNITED KINGDOM

## Abstract

**Background:**

Emerging data in chronic kidney disease (CKD) patients suggest that iron deficiency and higher circulating levels of erythropoietin (EPO) stimulate the expression and concomitant cleavage of the osteocyte-derived, phosphate-regulating hormone fibroblast growth factor 23 (FGF23), a risk factor for premature mortality. To date, clinical implications of iron deficiency and high EPO levels in the general population, and the potential downstream role of FGF23, are unclear. Therefore, we aimed to determine the associations between iron deficiency and higher EPO levels with mortality, and the potential mediating role of FGF23, in a cohort of community-dwelling subjects.

**Methods and findings:**

We analyzed 6,544 community-dwelling subjects (age 53 ± 12 years; 50% males) who participated in the Prevention of Renal and Vascular End-Stage Disease (PREVEND) study—a prospective population-based cohort study, of which we used the second survey (2001–2003)—and follow-up was performed for a median of 8 years. We measured circulating parameters of iron status, EPO levels, and plasma total FGF23 levels. Our primary outcome was all-cause mortality. In multivariable linear regression analyses, ferritin (ß = –0.43), transferrin saturation (TSAT) (ß = −0.17), hepcidin (ß = −0.36), soluble transferrin receptor (sTfR; ß = 0.33), and EPO (ß = 0.28) were associated with FGF23 level, independent of potential confounders. During median (interquartile range [IQR]) follow-up of 8.2 (7.7–8.8) years, 379 (6%) subjects died. In multivariable Cox regression analyses, lower levels of TSAT (hazard ratio [HR] per 1 standard deviation [SD], 0.84; 95% confidence interval [CI], 0.75–0.95; *P* = 0.004) and higher levels of sTfR (HR, 1.15; 95% CI 1.03–1.28; *P* = 0.01), EPO (HR, 1.17; 95% CI 1.05–1.29; *P* = 0.004), and FGF23 (HR, 1.20; 95% CI 1.10–1.32; *P* < 0.001) were each significantly associated with an increased risk of death, independent of potential confounders. Adjustment for FGF23 levels markedly attenuated the associations of TSAT (HR, 0.89; 95% CI 0.78–1.01; *P* = 0.06), sTfR (HR, 1.08; 95% CI 0.96–1.20; *P* = 0.19), and EPO (HR, 1.10; 95% CI 0.99–1.22; *P* = 0.08) with mortality. FGF23 remained associated with mortality (HR, 1.15; 95% CI 1.04–1.27; *P* = 0.008) after adjustment for TSAT, sTfR, and EPO levels. Mediation analysis indicated that FGF23 explained 31% of the association between TSAT and mortality; similarly, FGF23 explained 32% of the association between sTfR and mortality and 48% of the association between EPO and mortality (indirect effect *P* < 0.05 for all analyses). The main limitations of this study were the observational study design and the absence of data on intact FGF23 (iFGF23), precluding us from discerning whether the current results are attributable to an increase in iFGF23 or in C-terminal FGF23 fragments.

**Conclusions and relevance:**

In this study, we found that functional iron deficiency and higher EPO levels were each associated with an increased risk of death in the general population. Our findings suggest that FGF23 could be involved in the association between functional iron deficiency and increased EPO levels and death. Investigation of strategies aimed at correcting iron deficiency and reducing FGF23 levels is warranted.

## Abstract 2

Michele Eisenga and colleagues study the clinical consequences of, and biomarkers associated with, iron deficiency.

## Author summary

**Why was this study done?:**

- Iron deficiency, one of the most common nutritional disorders worldwide, is known to be associated with increased risk of death in the general population, although a clear mechanism has not been identified.
- In patient populations, it has been shown that higher levels of erythropoietin (EPO) are associated with adverse outcomes, but its significance in the general population has not been elucidated yet.
- Emerging data from the chronic kidney disease (CKD) field suggest that both iron deficiency and EPO lead to an up-regulated expression and concomitant cleavage of a phosphate-regulating hormone, fibroblast growth factor 23 (FGF23), another risk factor for mortality.

**What did the researchers do and find?:**

- We used the Prevention of Renal and Vascular End-Stage Disease (PREVEND) study, a prospective population-based cohort study from 2001 to 2003 involving 6,588 subjects and for which follow-up has been performed for a median of 8 years.
- We found that iron status parameters and EPO were the strongest determinants of FGF23 in the general population, even more than such well-described determinants as phosphate, kidney function, and calcium levels.
- Functional iron deficiency and EPO were associated with an increased risk of death in the general population, and FGF23 explained a substantial part of these associations.

**What do these findings mean?:**

- The current findings suggest that FGF23 is important in the pathophysiology of iron-deficiency–and EPO-mediated mortality in the population.
- This study provides the rationale to assess the potential benefits of iron supplementation to reduce mortality in the general population, possibly by lowering FGF23 levels.

## Introduction

Iron deficiency is one of the most common nutritional disorders worldwide [1]. In addition to its effect on quality of life and functional capacity, iron deficiency has previously been associated with an increased risk of all-cause and cardiovascular mortality in the general population [2]. However, the underlying mechanisms linking iron deficiency with mortality in this setting remain unknown. Emerging data from disease populations such as chronic kidney disease (CKD) patients suggest that iron deficiency stimulates the expression and concomitant cleavage of the osteocyte-derived, phosphate-regulating hormone fibroblast growth factor 23 (FGF23). Elevated levels of FGF23, in turn, have been shown to be associated with an increased risk of mortality in both community-dwelling individuals [3] and across stages of CKD [4–6].

Similarly, higher circulating endogenous erythropoietin (EPO) levels have been associated with an increased all-cause and cardiovascular mortality risk in various disease populations, including chronic heart failure patients, kidney transplant recipients, and elderly individuals [7–10]. In addition, higher doses of exogenous EPO increase the risk of cardiovascular events in CKD patients [11–13]. To date, it is unknown whether EPO levels are associated with adverse outcomes in the general population. Similar to iron deficiency, recent studies from our group and others have established that both endogenous and exogenous EPO may influence FGF23 production and metabolism in CKD patients [14–16].

Currently, the clinical implications of iron deficiency and high EPO levels in the general population, as well as the potential downstream role for FGF23, are unclear. Hence, in the current study, we investigated whether iron deficiency and EPO influence FGF23 levels, whether iron deficiency and elevated EPO levels are associated with an increased risk of death, and whether such associations could be explained by variation in FGF23 levels.

## Methods

### Study population

Details from the Prevention of Renal and Vascular End-Stage Disease (PREVEND) study have been described previously [17]. This study was planned following prior results indicating that iron deficiency and high EPO levels are associated with higher FGF23 levels and subsequently contribute to an increased mortality risk in kidney transplant recipients [16,18]. Between January and September 2018, we addressed the hypothesis that similar relationships would exist in the general population. For this study, we did not have a prespecified analysis plan, but we performed hypothesis-driven analyses in which no data-driven changes have taken place. This study is reported as per the Strengthening the Reporting of Observational Studies in Epidemiology (STROBE) guideline (S1 Checklist). In brief, from 1997 to 1998, all inhabitants of the city of Groningen, the Netherlands, who were 28 to 75 years old received a questionnaire on demographics, disease history, smoking habits, use of medication, and a vial to collect an early morning urinary sample (*n* = 85,421). Of these individuals, 40,856 (47.8%) responded. After exclusion of subjects with insulin-dependent diabetes mellitus and pregnant women, subjects with a urinary albumin concentration ≥10 mg/L (*n* = 6,000) and a randomly selected control group with a urinary albumin excretion <10 mg/L (*n* = 2,592) completed the screening protocol and, as such, formed the baseline PREVEND cohort (*n* = 8,592). For the current analyses, we used data from the second survey, which took place between 2001 and 2003 (*n* = 6,894), since for this visit, blood samples were also available. We excluded 436 subjects due to missing data on FGF23, resulting in 6,544 subjects (visually depicted in a flowchart in S1 Fig). The PREVEND study protocol was approved by the institutional medical review board and was in accordance with the Declaration of Helsinki. Written informed consent was obtained from all subjects.

### Exposures and outcomes

Fasting blood samples were drawn in the morning from all subjects from April 24, 2001, to December 3, 2003. All hematologic measurements were measured in fresh venous blood. Aliquots of these samples were stored immediately at −80°C until further analysis. Serum creatinine was measured using an enzymatic method on a Roche Modular analyzer (Roche Diagnostics, Mannheim, Germany). For predicting the estimated glomerular filtration rate (eGFR), the Chronic Kidney Disease Epidemiology Collaboration (CKD-EPI) was applied [19]. Serum iron was measured using a colorimetric assay, ferritin using immunoassay, and transferrin using an immunoturbidimetric assay (all Roche Diagnostics). Transferrin saturation (TSAT, %) was calculated as 100 × serum iron (μmol/L) ÷ 25 × transferrin (g/L) [20]. Serum EPO was measured using an immunoassay based on chemiluminescence (Immulite EPO assay, DPC, Los Angeles, CA). Serum hepcidin was measured with a competitive enzyme-linked immunosorbent assay (ELISA), as described elsewhere with intra- and interassay coefficients of variation (CVs) of 8.6% and 16.2%, respectively [21]. Soluble transferrin receptor (sTfR) was measured using an automated homogenous immunoturbidimetric assay with intra- and interassay CVs <2% and <5% [22]. Total FGF23 levels were measured in plasma EDTA samples with a human FGF23 ELISA (Quidel Corp., San Diego, CA) directed against 2 different epitopes within the C-terminal part of the FGF23 molecule. Hence, the assay measures both the intact hormone and the C-terminal fragments and, as such, measures total FGF23 levels. In our hands, this ELISA had intra- and interassay CVs of <5% and <16% in blinded replicated samples, respectively [23]. We assessed prospective associations of iron status parameters, EPO, hepcidin, and FGF23 levels with death. In the PREVEND cohort, data on mortality were received through the municipal register, and follow-up was available until January 1, 2011.

### Statistical analyses

Data were analyzed using IBM SPSS software, version 23.0 (SPSS, Chicago, IL), R version 3.2.3 (Vienna, Austria), and STATA 14.1 (STATA Corp., College Station, TX). Baseline characteristics are described as means with standard deviation (SD) for normally distributed variables, as medians with interquartile range (IQR) for skewed variables, or as numbers with corresponding percentages for categorical variables. Differences in baseline characteristics across tertiles of FGF23 were tested with a one-way ANOVA for normally distributed variables, Kruskal-Wallis test for skewed variables, and a chi-squared test for categorical variables. Hereafter, we assessed the relationship between iron status parameters and EPO as determinants of FGF23 by calculating Spearman correlation coefficients. We subsequently performed multivariable analyses adjusted for age, sex, eGFR, urinary albumin excretion, systolic blood pressure, alcohol use (5 categories), smoking status (never, former, or current smoker), hemoglobin, mean corpuscular volume (MCV), and plasma/serum levels of high-sensitivity C-reactive protein (hs-CRP), phosphate, calcium, 25-hydroxyvitamin D (25D), and parathyroid hormone (PTH). In all analyses, skewed variables, i.e., FGF23, ferritin, hepcidin, sTfR, EPO, urinary albumin excretion, PTH, and hs-CRP, were naturally log transformed prior to inclusion. Second, we performed stepwise backward multivariable linear regression analyses in which we determined whether iron status parameters and EPO remained major determinants of FGF23 levels. Of note, because the different iron status parameters are highly correlated with each other (S1 Table), we included all iron status parameters individually in multivariable linear regression analyses. To visualize the cross-sectional associations between the different iron status parameters—EPO and FGF23—plots were generated using locally weighted scatterplot smoothing.

We subsequently assessed the associations of iron status parameters and EPO levels with all-cause mortality using Cox proportional hazard regression analyses. Assumption of proportionality in Cox regression analyses was checked using Schoenfeld residuals plots. We constructed multivariable models adjusted for age and sex (model 1), and additionally for eGFR, urinary albumin excretion, body mass index (BMI), systolic blood pressure, hs-CRP, presence of diabetes, smoking, alcohol use, and use of antihypertensives (model 2). Subsequently, we adjusted for FGF23 (model 3). Similarly, we assessed whether FGF23 levels were associated with mortality, independent of potential confounders, including adjustment for iron status parameters and EPO in the final models. Finally, we performed mediation analyses with the methods described by Preacher and Hayes, which is based on logistic regression. These analyses allow for testing significance and magnitude of mediation [24,25]. Overall, 3.4% of demographic data were missing and were imputed using regressive switching [26]. Five datasets were multiply imputed, and results were pooled according to Rubin’s rules [27]. In all analyses, a two-sided significance level <0.05 was considered significant.

## Results

### Baseline characteristics

We included 6,544 community-dwelling subjects (age 53 ± 12 years; 50% males) with a median (IQR) FGF23 level of 70 (57–87) RU/mL. Mean hemoglobin concentration was 13.7 ± 1.2 g/dL; median ferritin concentration was 96 (47–172) μg/L; mean TSAT was 25.0 ± 9.5%; median sTfR level was 2.5 (2.1–3.0) mg/L; median hepcidin level was 8.5 (4.6–13.8) ng/L; and median EPO level was 7.8 (5.9–10.3) IU/L. Iron deficiency based on ferritin levels (i.e., ferritin < 15 μg/L for women and < 30 μg/L for men) was present in 448 (7%) individuals [28]. Iron deficiency based on low TSAT levels (i.e., TSAT < 20%) was present in 1,806 (28%) individuals [2]. EPO-stimulating agents (ESAs) and oral or intravenous iron were not used by any of the subjects. Across tertiles of FGF23, we observed inverse associations with ferritin, TSAT, and hepcidin and a positive association with sTfR and EPO levels. Additional demographic, clinical, and laboratory parameters are depicted in Table 1.

**Table 1:** Baseline characteristics of 6,544 community-dwelling subjects according to tertiles of total FGF23 levels.

		Tertiles of total FGF23 (RU/mL)			
Baseline characteristic	All patients (*n* = 6,544)	T1 (*n* = 2,177)	T2 (*n* = 2,186)	T3 (*n* = 2,181)	*P* value
		[20.7–60.6]	[60.7–80.2]	[80.3–3,494.6]	
**Age (y)**	53 ± 12	51 ± 12	53 ± 12	55 ± 12	<0.001
**Male sex (*n*, %)**	3,251 (50)	1,193 (55)	1,152 (53)	906 (42)	<0.001
**BMI, kg/m**^**2**^	26.7 ± 4.4	26.1 ± 3.8	26.6 ± 4.3	27.5 ± 4.8	<0.001
**Alcohol use**					<0.001
No alcohol use (*n*, %)	1,722 (26)	482 (22)	534 (24)	706 (32)	
1–4 units/mo (*n*, %)	1,093 (17)	386 (18)	360 (17)	347 (16)	
2–7 units/wk (*n*, %)	2042 (31)	730 (34)	675 (31)	637 (29)	
>1–3 units/d (*n*, %)	1,416 (22)	498 (23)	515 (24)	403 (19)	
>3 units/d (*n*, %)	271 (4)	81 (4)	102 (5)	88 (4)	
**Smoking status**					<0.001
Never smoker (*n*, %)	1,967 (30)	756 (35)	647 (30)	564 (26)	
Former smoker (*n*, %)	2,748 (42)	957 (44)	945 (43)	846 (39)	
Current smoker (*n*, %)	1,829 (28)	464 (21)	594 (27)	771 (35)	
Diabetes mellitus (*n*, %)	403 (6)	95 (4)	118 (5)	190 (9)	<0.001
**Systolic blood pressure (mmHg)**	126 ± 19	125 ± 18	126 ± 19	128 ± 20	0.001
**Diastolic blood pressure (mmHg)**	73 ± 9	73 ± 9	73 ± 9	74 ± 9	0.90
**Laboratory measurements**					
***Iron status***					
Ferritin (μg/L)	96 (47–172)	110 (61–190)	102 (53–178)	74 (28–149)	<0.001
TSAT (%)	25.0 ± 9.5	26.8 ± 9.3	25.9 ± 9.1	22.5 ± 9.5	<0.001
sTfR (mg/L)	2.5 (2.1–3.0)	2.4 (2.1–2.8)	2.4 (2.1–2.9)	2.7 (2.2–3.3)	<0.001
Hepcidin (ng/mL)	8.5 (4.6–13.8)	9.4 (5.5–14.5)	8.9 (5.2–13.9)	7.1 (2.9–12.8)	<0.001
EPO (IU/L)	7.8 (5.9–10.3)	7.2 (5.6–9.3)	7.7 (5.9–9.9)	8.6 (6.4–11.8)	<0.001
Hemoglobin (g/dL)	13.7 ± 1.2	13.8 ± 1.1	13.8 ± 1.1	13.6 ± 1.4	<0.001
MCV (fL)	90 ± 5	91 ± 4	91 ± 4	90 ± 6	<0.001
Total cholesterol (mg/dL)	209.6 ± 40.4	208.5 ± 40.2	209.8 ± 40.4	210.4 ± 40.6	0.17
Glucose (mg/dL)	91 ± 21	89 ± 18	90 ± 20	93 ± 24	<0.001
Phosphate (mg/dL)	3.13 ± 0.87	3.07 ± 0.89	3.10 ± 0.74	3.21 ± 0.95	<0.001
Calcium (mg/dL)	9.23 ± 0.46	9.19 ± 0.44	9.24 ± 0.47	9.26 ± 0.47	0.18
PTH (pg/mL)	46.0 (38.4–55.3)	44.9 (37.5–53.5)	45.5 (38.4–54.4)	47.5 (39.2–57.6)	<0.001
25 (OH) vitamin D (ng/mL)	22.8 ± 10.0	23.4 ± 10.2	23.0 ± 9.9	21.9 ± 10.0	<0.001
eGFR (ml/min/1.73 m^2^)	91.8 ± 17.2	98.5 ± 14.6	92.4 ± 15.4	85.6 ± 18.7	<0.001
Creatinine (mg/dL)	0.96 ± 0.24	0.93 ± 0.15	0.96 ± 0.16	0.99 ± 0.35	<0.001
Urinary albumin excretion (mg/24 h)	8.7 (6.1–16.0)	8.4 (6.0–14.6)	8.5 (6.1–14.7)	9.5 (6.2–20.3)	<0.001
hs-CRP (mg/L)	1.4 (0.6–3.1)	1.1 (0.5–2.5)	1.3 (0.6–2.9)	1.7 (0.8–3.9)	<0.001
**Medication use**					
Antihypertensives* (*n*, %)	1,299 (20)	269 (12)	397 (18)	633 (29)	<0.001
ACE inhibitors or AII antagonists (*n*, %)	580 (9)	126 (6)	198 (9)	256 (12)	<0.001

*Includes ACE or AII antagonists; *P* for trend was calculated with linear regression for continuous variables and with chi-squared test for dichotomous and categorical variables. SI conversion factors: to convert hepcidin from ng/mL to nmol/L, divide by 2.789; to convert hemoglobin from g/dL to mmol/L, multiply by 0.6206; to convert total cholesterol from mg/dL to mmol/L, multiply by 0.0259; to convert glucose from mg/dL to mmol/L, multiply by 0.0555; to convert phosphate from mg/dL to mmol/L, multiply by 0.3229; to convert calcium from mg/dL to mmol/L, multiply by 0.2495; to convert PTH from pg/mL to pmol/L, multiply by 0.105; to convert vitamin D from ng/mL to nmol/L, multiply by 2.496; to convert creatinine from mg/dL to μmol/L, multiply by 88.42.

**Abbreviations:** ACE, angiotensin converting enzyme; BMI, body mass index; CRP, C-reactive protein; eGFR, estimated glomerular filtration rate; EPO, erythropoietin; FGF23, fibroblast growth factor 23; hs-CRP, high-sensitivity C-reactive protein; MCV, mean corpuscular volume; PTH, parathyroid hormone; SI, system of units; sTfR, soluble transferrin receptor; TSAT, transferrin saturation.

### Iron parameters, EPO, and FGF23

**Fig 1:**
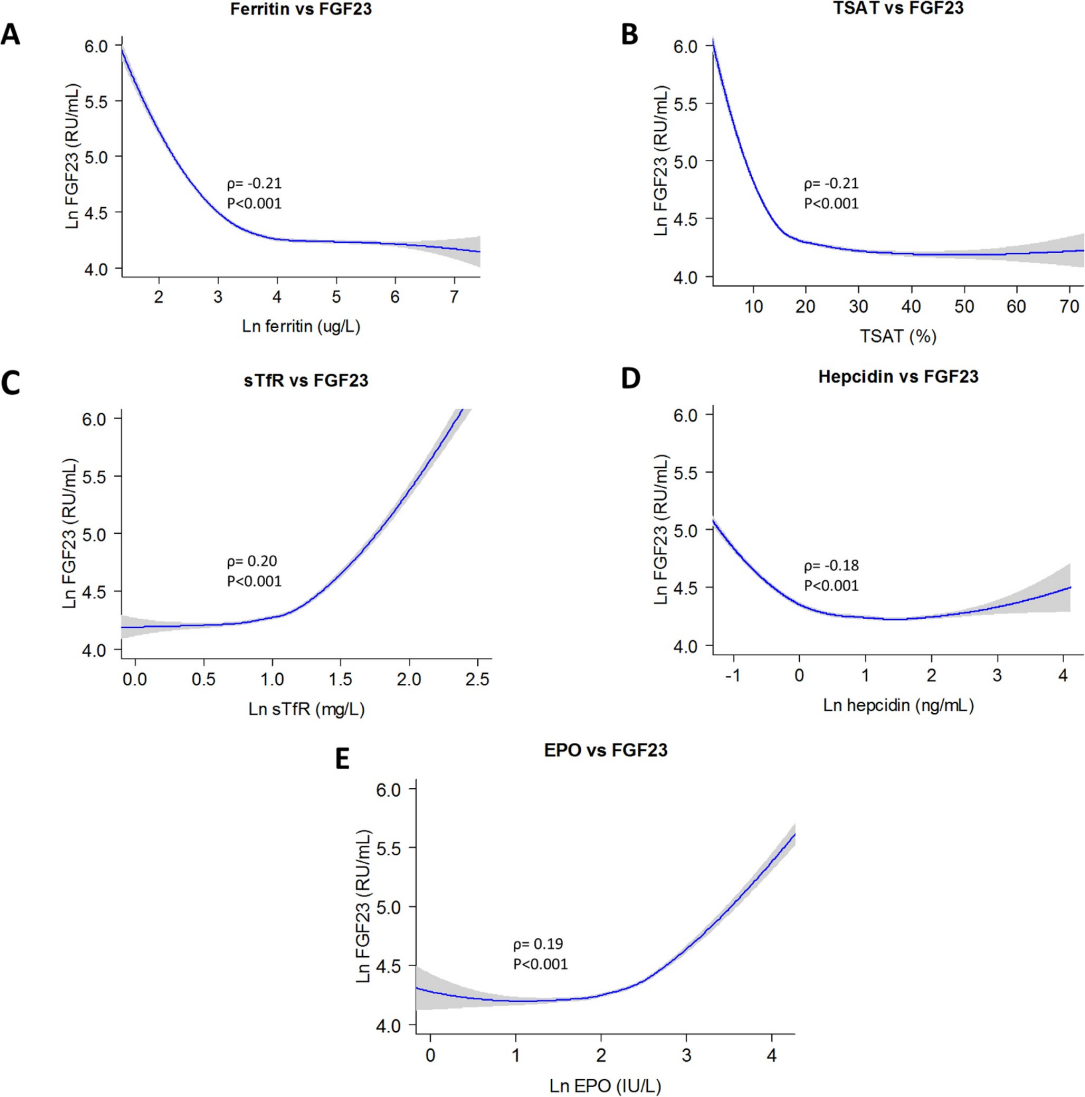
Cross-sectional associations of iron status parameters and EPO with FGF23. Plots were generated with use of locally weighted scatterplot smoothing and show the association of FGF23 with ferritin (Panel A), TSAT (Panel B), sTfR (Panel C), hepcidin (Panel D), and EPO (Panel E). Lines and band represent means and 95% CIs, respectively. CI, confidence interval; FGF23, ferritin, sTfR, hepcidin, and EPO were naturally log transformed. EPO, erythropoietin; FGF23, fibroblast growth factor 23; sTfR, soluble transferrin receptor; TSAT, transferrin saturation.

In univariate analyses, higher FGF23 levels correlated with lower levels of ferritin (ρ = –0.21, *P* < 0.001), TSAT (ρ = –0.21, *P* < 0.001), sTfR (ρ = 0.20, *P* < 0.001), and hepcidin (ρ = –0.18, *P* < 0.001) and with higher levels of serum EPO (ρ = 0.19, *P* < 0.001) (Fig 1A–1E). After adjustment for age, sex, eGFR, urinary albumin excretion, systolic blood pressure, alcohol use, smoking status, hemoglobin, MCV, hs-CRP, phosphate, calcium, 25D, and PTH, all iron parameters—including ferritin (ß = –0.43, *P <* 0.001), TSAT (ß = –0.17, *P* < 0.001), sTfR (ß = 0.33, *P <* 0.001), and hepcidin (ß = –0.36, *P* < 0.001)—remained strongly and independently associated with FGF23 levels. Similarly, EPO (ß = 0.28, *P* < 0.001) remained independently associated with FGF23. In stepwise backward linear regression analyses, ferritin (ß = –0.38, *P <* 0.001), sTfR (ß = 0.27, *P <* 0.001), hepcidin (ß = –0.31, *P <* 0.001), and EPO (ß = 0.21, *P <* 0.001) levels were identified as the strongest determinants of FGF23 levels, with higher standardized regression coefficients than more established determinants of FGF23, including eGFR (ß = –0.20, *P <* 0.001), phosphate (ß = 0.13, *P <* 0.001), and calcium (ß = 0.17, *P <* 0.001) (S2 Table).

### Iron status and mortality

During a median (IQR) follow-up of 8.2 (7.7–8.8) years, 379 (6%) subjects died. After 1, 5, and 8 years of follow-up, 6,437, 6,040, and 3,993 subjects, respectively, were still followed up in the study. We assessed the associations of the individual iron status parameters, EPO and FGF23, with mortality (Table 2). After adjustment for age and sex, neither ferritin nor hepcidin was associated with risk of death (hazard ratio [HR] per 1 SD higher ln[ferritin], 0.95; 95% confidence interval [CI], 0.84–1.07; *P* = 0.38; HR per 1 SD higher ln[hepcidin], 0.99; 95% CI 0.88–1.11; *P* = 0.80, respectively). In contrast, a lower TSAT was strongly associated with an increased risk of death (age- and sex-adjusted HR per 1 SD higher TSAT, 0.79; 95% CI 0.70–0.88; *P* < 0.001). After further adjustment for eGFR, urinary albumin excretion, BMI, systolic blood pressure, hs-CRP, presence of diabetes, smoking, alcohol use, and use of antihypertensives (model 2), the association between TSAT and mortality persisted (HR, 0.84; 95% CI 0.75–0.95; *P* = 0.004). However, subsequent adjustment for FGF23 attenuated the association, such that TSAT was no longer significantly associated with death (HR, 0.89; 95% CI 0.78–1.01; *P* = 0.06).

**Table 2:** HRs (and 95% CIs) for death according to FGF23, iron status parameters, and EPO.

Biomarker	Model 1	Model 2	Model 3
**FGF23**	1.29 (1.20–1.34)	1.20 (1.10–1.32)	1.15 (1.04–1.27)
**EPO**	1.22 (1.10–1.34)	1.17 (1.05–1.29)	1.10 (0.99–1.22)
**TSAT**	0.79 (0.70–0.88)	0.84 (0.75–0.95)	0.89 (0.78–1.01)
**sTfR**	1.17 (1.06–1.30)	1.15 (1.03–1.28)	1.08 (0.96–1.20)
**Ferritin**	0.95 (0.84–1.07)	0.91 (0.80–1.03)	1.02 (0.90–1.16)
**Hepcidin**	0.99 (0.88–1.11)	0.95 (0.85–1.07)	1.03 (0.91–1.16)

The table shows the different HRs for death according to the different natural log-transformed (except TSAT) parameters standardized to 1 SD. Model 1 is adjusted for age and sex; model 2 is adjusted for eGFR, urinary albumin excretion, BMI, systolic blood pressure, hs-CRP, presence of diabetes, smoking, alcohol use, and use of antihypertensives; for FGF23, extra adjustments for calcium, phosphate, 25 vitamin D, and PTH. Model 3 has been adjusted for FGF23 in the EPO and iron analyses, whereas it has been adjusted for EPO, TSAT, and sTfR in the FGF23 analysis.

**Abbreviations:** CI, confidence interval; eGFR, estimated glomerular filtration rate; EPO, erythropoietin; FGF23, fibroblast growth factor 23; HR, hazard ratio; hs-CRP, high-sensitivity C-reactive protein; PTH, parathyroid hormone; SD, standard deviation; sTfR, soluble transferrin receptor; TSAT, transferrin saturation.

Similarly, a higher plasma sTfR level, indicating functional iron deficiency, was associated with a higher risk of mortality in a model adjusted for age and sex (HR per 1 SD higher ln[sTfR], 1.17; 95% CI 1.06–1.30; *P* = 0.004). In multivariable analyses (model 2), the association between sTfR and mortality remained significant (HR, 1.15; 95% CI 1.03–1.28; *P* = 0.01). However, adjustment for FGF23 strongly attenuated the association, rendering the association between sTfR and mortality nonsignificant (HR, 1.08; 95% CI 0.96–1.20; *P* = 0.19).

In mediation analyses, we analyzed whether the significant associations between iron status parameters (i.e., TSAT and sTfR) and mortality were mediated by FGF23 (Table 3). FGF23 was identified as a significant mediator (indirect effect *P* < 0.05; 31% of the association between TSAT and mortality was explained by FGF23 and 32% of the association between sTfR and mortality).

**Table 3:** Mediation analyses of FGF23 on the association between TSAT, sTfR, EPO, and mortality in the general population.

Independent variable	Potential mediator	Outcome	Effect (path)*	Multivariable model**	
					Coefficient (95% CI, bc)***	Proportion mediated†
**TSAT**	**FGF23**	**Mortality**	Indirect effect (*ab* path)	−0.030 (−0.050 to −0.012)	**31%**
			Total effect (*ab* + *c*’ path)	−0.098 (−0.174 to −0.026)	
**sTfR**	**FGF23**	**Mortality**	Indirect effect (*ab* path)	0.039 (0.009 to 0.072)	**32%**
			Total effect (*ab* + *c*’ path)	0.123 (0.040 to 0.205)	
**EPO**	**FGF23**	**Mortality**	Indirect effect (*ab* path)	0.034 (0.012 to 0.060)	**48%**
			Total effect (*ab* + *c*’ path)	0.071 (0.008 to 0.139)	
**EPO**	**TSAT**	**FGF23**	Indirect effect (*ab* path)	0.037 (0.028 to 0.047)	**12%**
			Total effect (*ab* + *c*’ path)	0.318 (0.275 to 0.365)	
**EPO**	**sTfR**	**FGF23**	Indirect effect (*ab* path)	0.108 (0.086 to 0.133)	**33%**
			Total effect (*ab* + *c*’ path)	0.326 (0.277 to 0.379)	

*The coefficients of the indirect *ab* path and the total *ab* + *c’* path are standardized for the SDs of TSAT, sTfR, EPO, FGF23, and all-cause mortality.

**All coefficients are adjusted for age, sex, eGFR, urinary albumin excretion, BMI, systolic blood pressure, hs-CRP, presence of diabetes, smoking, alcohol use, and use of antihypertensives.

***95% CIs for the indirect and total effects were bias-corrected CIs after running 2,000 bootstrap samples.

†The size of the significant mediated effect is calculated as the standardized indirect effect divided by the standardized total effect multiplied by 100.

**Abbreviations:** bc, bias corrected; BMI, body mass index; CI, confidence interval; EPO, erythropoietin; eGFR, estimated glomerular filtration rate; FGF23, fibroblast growth factor 23; hs-CRP, high-sensitivity C-reactive protein; SD, standard deviation; sTfR, soluble transferrin receptor; TSAT, transferrin saturation.

### EPO and mortality

In age- and sex-adjusted analyses, higher serum EPO levels were associated with an increased risk of death (HR per 1 SD higher ln[EPO], 1.22; 95% CI 1.10–1.34; *P* < 0.001, Table 2). In multivariable analyses (model 2), the association between EPO and mortality persisted (HR, 1.17; 95% CI 1.05–1.29; *P* = 0.004). However, adjustment for FGF23 abrogated the association between EPO and mortality, rendering the association nonsignificant (HR, 1.10; 95% CI 0.99–1.22; *P* = 0.08).

In mediation analyses, we analyzed whether the significant association between EPO and mortality was mediated by FGF23 (Table 3). FGF23 was identified as a significant mediator (indirect effect *P* < 0.05; 48% of the association between EPO and mortality was explained by FGF23). Because functional iron deficiency often occurs in states of EPO-mediated erythropoiesis, we also analyzed whether the positive association between EPO and FGF23 might be, at least in part, mediated by TSAT and sTfR. Both parameters were found to be significant mediators in the positive association between EPO and FGF23 (indirect effect *P* < 0.05; 12% by TSAT and 33% by sTfR, independent of potential confounders, Table 3).

### FGF23 and mortality

In age- and sex-adjusted analyses, higher plasma FGF23 levels were strongly associated with an increased risk of death (HR per 1 SD higher ln[FGF23], 1.29; 95% CI 1.20–1.34; *P* < 0.001, Table 2). In multivariable analyses (model 2) with inclusion of bone mineral parameters (i.e., calcium, phosphate, 25D, and PTH), the association between FGF23 and mortality remained (HR, 1.20; 95% CI 1.10–1.32; *P* < 0.001, Table 2). Further adjustment for TSAT, sTfR, and EPO did not materially change the association between FGF23 and mortality (HR, 1.15; 95% CI 1.04–1.27; *P* = 0.008).

## Discussion

In this study, we found that markers of iron deficiency, especially lower iron availability (i.e., lower TSAT and higher sTfR), as well as elevated serum EPO, were associated with an increased risk of mortality in community-dwelling individuals. Notably, we identified FGF23 as a potential mediator of iron-deficiency–and EPO-related mortality. Iron deficiency and elevated levels of EPO were major determinants of FGF23 levels, to a greater extent than more established determinants such as renal function and serum calcium, PTH, and phosphate. FGF23 in itself was strongly associated with mortality independent of adjustment for iron status parameters and EPO. Our findings suggest that FGF23 could be involved in the pathophysiology of iron-deficiency–and EPO-mediated mortality in the population.

We first addressed the relationship between iron deficiency, measured by 4 different parameters, and all-cause mortality in the general population. Our results are consistent with a previous study identifying low TSAT as a predictor of mortality in the general population [2]. Of note, the prospective associations with mortality differed among the various iron parameters. While TSAT and sTfR were strongly associated with mortality, ferritin and hepcidin were not. This discrepancy is most likely explained by the fact that these markers reflect different aspects of iron metabolism. Serum ferritin is a surrogate for body iron stores, but as an acute-phase reactant, it is also strongly up-regulated by inflammation, malignancy, and alcohol intake. Hepcidin is also an acute-phase reactant and is highly correlated with serum ferritin. In contrast, TSAT is more a marker of iron availability for erythropoiesis. Elevated levels of sTfR reflect an increased tissue iron demand, but not body iron stores, and are less affected by concomitant chronic disease and inflammation [29]. In the setting of increased metabolic requirements for iron, transferrin receptors are overexpressed on erythroid precursors in the bone marrow and are shed, resulting in increased sTfR levels in the circulation. Hence, an increased sTfR level reflects both erythroid activity and functional iron deficiency. Because functional iron deficiency occurs in patients with significant EPO-mediated erythropoiesis or as a response to treatment with ESAs [30], we also aimed to assess the association between endogenous EPO levels, as a reflection of tissue hypoxia, and mortality. Prior studies conducted in various populations, including in elderly individuals, kidney transplant recipients, and in patients with chronic heart failure, found that higher EPO levels are associated with an increased risk of death, even independent of hemoglobin levels [8–10]. Furthermore, large randomized trials in chronic heart failure and CKD patients striving for stronger correction of anemia with ESAs were associated with an increased risk of mortality [12,13,31,32]. In the current study, we identified for the first time a strong and independent association between higher serum EPO levels and increased mortality in community-dwelling individuals.

The associations we observed between functional iron deficiency, high EPO levels, and mortality led us to explore FGF23 as a potential downstream factor mediating these associations, given accumulating evidence supporting a direct relationship between iron status, EPO, and FGF23 metabolism [16,18,33–35]. Recently, our group and others demonstrated that iron deficiency is a strong determinant of total FGF23 levels in CKD and kidney transplant recipients [18,36]. Mechanistically, it has recently been shown that iron deficiency stabilizes hypoxia-inducible factor 1-alpha, which in turn up-regulates furin, promoting cleavage of the intact FGF23 (iFGF23) molecule into C-terminal FGF23 fragments [34,37,38]. Furthermore, EPO-induced up-regulation of FGF23 production has been demonstrated in CKD patients, kidney transplant recipients, and in animal models in which circulating EPO levels are elevated due to either endogenous or exogenous sources [14,33]. Currently, the exact mechanism by which EPO increases bone and bone marrow FGF23 transcription and FGF23 post-translational cleavage is unknown; however, Rabadi and colleagues observed in bled mice decreased polypeptide N-acetylgalactosaminyltransferase 3 (GalNT3) bone marrow mRNA expression, which protects iFGF23 from proteolysis by furin, allowing increased FGF23 cleavage [14]. Our group, together with collaborators, recently also showed that in mice with chronically elevated endogenous EPO levels, a decreased GalNT3 bone marrow mRNA expression was present, without differences in family with sequence similarity 20, member C (Fam20C) or furin expression [16]. In the current study, multivariable cross-sectional analyses also demonstrated strong associations of iron parameters and EPO with FGF23, independent of more established determinants of FGF23, including serum phosphate and eGFR.

We subsequently found that the associations between functional iron deficiency—reflected by low TSAT or high sTfR levels—and mortality were mediated by FGF23. Moreover, EPO-related mortality was also for a considerable part explained by variation in FGF23 levels. Of interest, the positive association between EPO and FGF23 was in part mediated by functional iron deficiency. These findings support our hypothesis that FGF23 is closely related to erythropoiesis and that up-regulation of FGF23 induced by iron deficiency or high EPO levels may subsequently lead to a higher mortality risk. The association between FGF23 and death has been previously demonstrated in different patient populations, including CKD, renal transplant recipients, acute kidney injury, chronic heart failure, and in the general population [3,4,39,40]. The downstream consequences of elevated levels of FGF23 have not been fully elucidated yet. Many reports have revealed that iFGF23 has biologic activity through binding to several FGF23 receptors, including FGFR1, FGFR2, and FGFR4. Besides the classic functions of iFGF23 in regulating renal phosphate handling and vitamin D metabolism, recent studies have demonstrated several “off-target” effects of iFGF23. Preclinical studies demonstrated that FGF23 can induce left ventricular hypertrophy by binding to FGF23 receptor 4 in cardiac myocytes, and promote endothelial dysfunction [41,42]. Furthermore, FGF23 stimulates fibrosis in the kidneys [43], exerts proinflammatory effects by up-regulation of interleukin-6 production [44], and impairs immune function [45]. Finally, it is known that abnormal FGF signaling can promote tumor development by stimulating cancer cell proliferation, invasion, and survival and by supporting tumor angiogenesis [46,47]. Feng and colleagues showed that exogenous FGF23 promotes prostate cancer proliferation, invasion, and independent growth in vitro, whereas FGF23 knockdown in model systems decreased tumor progression both in vitro and in vivo [48]. Given the strong and independent association of FGF23 with mortality and the emerging pathophysiological implications of FGF23, it seems important to unravel major determinants of FGF23 in order to be able to reduce FGF23 levels. In the current study, we have shown that, in the general population, iron deficiency is a major determinant of FGF23 levels, implicating that iron deficiency could potentially be an easily modifiable driver of high FGF23 levels. The potential benefits of iron supplementation to reduce mortality in the general population remain to be addressed in prospective studies.

Our study has several strengths as well as limitations. Major strengths include the availability of FGF23 along with multiple iron status parameters (including hepcidin and sTfR) and EPO in a large population-based cohort. On the other hand, we were unable to measure iFGF23 levels, because samples were not stored with protease inhibitors, and iFGF23 has been shown to be susceptible to degradation with long-term storage [49]. This precludes us from discerning whether the elevated levels of total FGF23 that we observed are attributable to increased circulating levels of intact, biologically active FGF23 or due to increased levels of C-terminal fragments, which are not biologically active. The latter could be due to increased production of FGF23 matched by a concomitant increase in FGF23 cleavage. This pattern has been observed in previous studies in various disease populations, and we speculate that the same pattern might occur in the general population as well. Furthermore, due to the observational design, residual confounding might have biased the results despite the substantial number of potentially confounding factors for which we adjusted. For example, it is known that TSAT is also influenced by inflammation (similar to ferritin and hepcidin); it might be that, despite adjustment for hs-CRP, inflammation has influenced this specific association. Finally, although we have strong evidence from the literature that FGF23 is a mediator in the association of iron deficiency and EPO with mortality, we cannot exclude that an unmeasured cause of mortality or alternative potential mediators have influenced the current results.

In conclusion, we have shown that functional iron deficiency and higher EPO levels are strongly associated with increased all-cause mortality in the general population. Furthermore, we demonstrated that the increased mortality risk in individuals with diminished iron availability or increased levels of EPO seems to be substantially attributable to variation in FGF23 levels. Future studies will be needed to delineate in more detail the underlying mechanism for the currently identified associations.

## Supporting information

S1 Checklist STROBE checklist. STROBE, Strengthening the Reporting of Observational Studies in Epidemiology.
(DOCX)

S1 Fig Flowchart of the included 6,544 subjects of the PREVEND study. PREVEND, Prevention of Renal and Vascular End-Stage Disease.
(DOCX)

S1 Table Correlation matrix between the different iron status parameters. The table shows the Pearson correlation coefficients. ***P < 0.001.
(DOCX)

S2 Table Determinants of FGF23 levels in the general population. Iron parameters have been placed separately in multivariable analyses; all reported coefficients of the other variables are from the multivariable model, including ferritin. FGF23, fibroblast growth factor 23.
(DOCX)

## Abbreviations

25D: 25-hydroxyvitamin D
ACE: angiotensin converting enzyme
BMI: body mass index
CI: confidence interval
CKD: chronic kidney disease
CKD-EPI: Chronic Kidney Disease Epidemiology Collaboration
CV: coefficient of variation
eGFR: estimated glomerular filtration rate
ELISA: enzyme-linked immunosorbent assay
EPO: erythropoietin
ESA: EPO-stimulating agent
Fam20C: family with sequence similarity 20, member C
FGF23: fibroblast growth factor 23
GALNT3: N-acetylgalactosaminyltransferase 3
HR: hazard ratio
hs-CRP: high-sensitivity C-reactive protein
iFGF23: intact FGF23
IQR: interquartile range
MCV: mean corpuscular volume
PREVEND: Prevention of Renal and Vascular End-Stage Disease
PTH: parathyroid hormone
SD: standard deviation
SI: system of units
sTfR: soluble transferrin receptor
STROBE: Strengthening the Reporting of Observational Studies in Epidemiology
TSAT: transferrin saturation
