# Supplementary figures and images for: Iron deficiency, elevated erythropoietin, fibroblast growth factor 23, and mortality in the general population of the Netherlands: A cohort study
Source: PLoS Med. 2019 Jun 6;16(6):e1002818. doi: 10.1371/journal.pmed.1002818 (PMC6553711; doi:10.1371/journal.pmed.1002818)

**S1_Fig**. Flowchart ofthe included 6,544 subjects of the PREVEND study


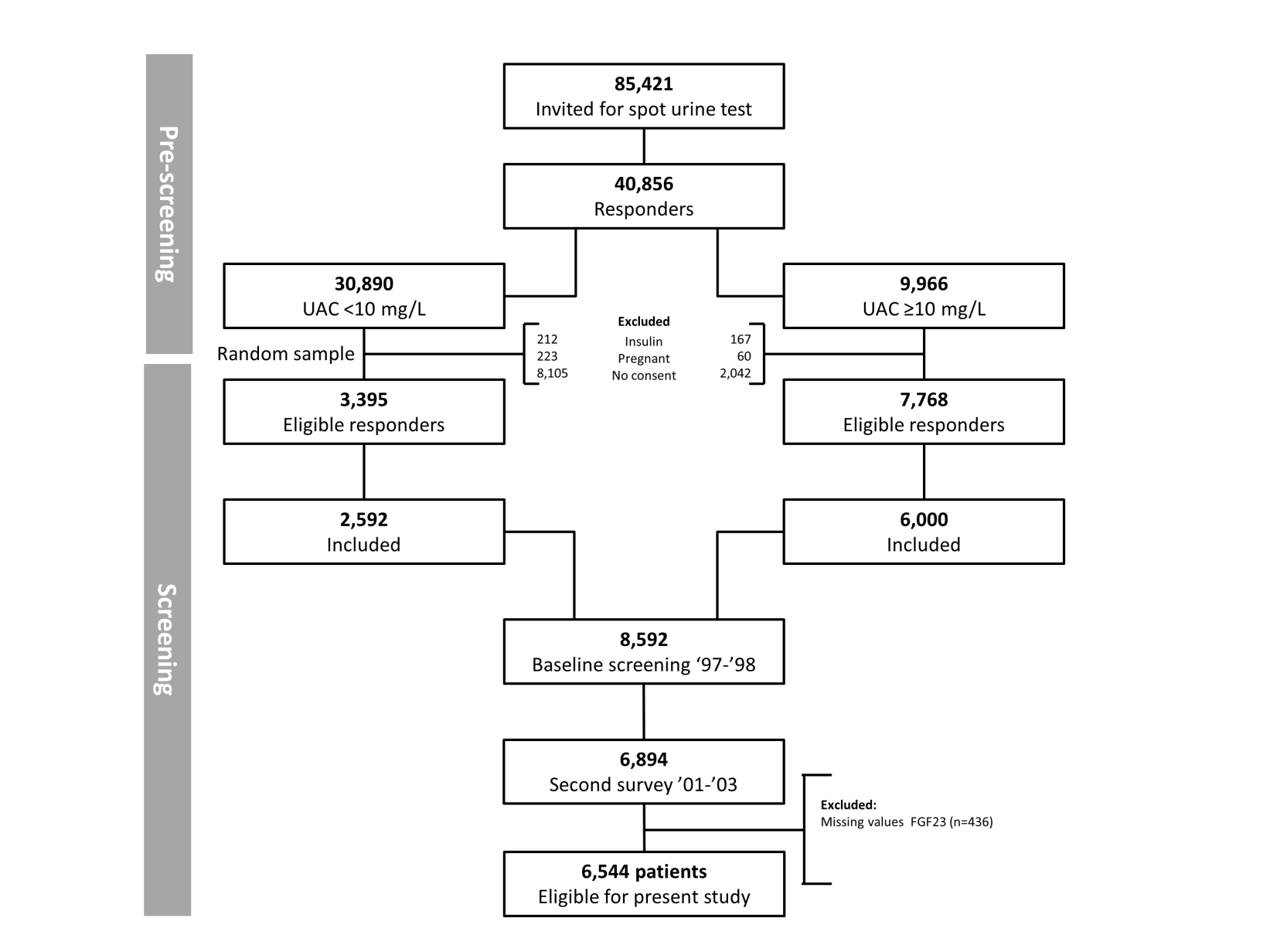

Supplement: S1 Fig — PREVEND, Prevention of Renal and Vascular End-Stage Disease. (DOCX) [file pmed.1002818.s002.docx]
