# Supplementary material for: Iron deficiency, elevated erythropoietin, fibroblast growth factor 23, and mortality in the general population of the Netherlands: A cohort study
Source: PLoS Med. 2019 Jun 6;16(6):e1002818. doi: 10.1371/journal.pmed.1002818 (PMC6553711; doi:10.1371/journal.pmed.1002818)
Supplement: S1 Table — The table shows the Pearson correlation coefficients. ***P < 0.001. (DOCX) [file pmed.1002818.s003.docx]

**S1_Table.** Correlation matrix between the different iron status parameters

|  |  | **Ferritin** | **Hepcidin** |  | **TSAT** | **sTfR** |
| --- | --- | --- | --- | --- | --- | --- |
| **Ferritin** |  | 1.00^***^ |  |  |  |  |
| **Hepcidin** |  | 0.77^***^ | 1.00^***^ |  |  |  |
| **TSAT** |  | 0.32^***^ | 0.27^***^ |  | 1.00^***^ |  |
| **sTfR** |  | -0.14^***^ | -0.13^***^ |  | -0.33^***^ | 1.00^***^ |

The table shows the Pearson correlation coefficients. ^***^ P<0.001
