# Supplementary material for: Iron deficiency, elevated erythropoietin, fibroblast growth factor 23, and mortality in the general population of the Netherlands: A cohort study
Source: PLoS Med. 2019 Jun 6;16(6):e1002818. doi: 10.1371/journal.pmed.1002818 (PMC6553711; doi:10.1371/journal.pmed.1002818)
Supplement: S2 Table — Iron parameters have been placed separately in multivariable analyses; all reported coefficients of the other variables are from the multivariable model, including ferritin. FGF23, fibroblast growth factor 23. (DOCX) [file pmed.1002818.s004.docx]

**S2_Table.** Determinants of FGF23 levels in the general population

| **Parameter** | **Univariate analysis** | | | **Multivariate analysis** | | |
| --- | --- | --- | --- | --- | --- | --- |
|  | **std. ß p-value** | | | **std. ß p-value** | | |
| **Demographics** |  |  |  |  |  |  |
| Age (yrs) | 0.10 |  | <0.001 |  |  |  |
| Sex (male vs. female) | -0.12 |  | <0.001 | 0.19 |  | <0.001 |
| BMI (kg/m^2^) | 0.10 |  | <0.001 | 0.13 |  | <0.001 |
| Systolic blood pressure (mmHg) | 0.04 |  | 0.001 |  |  |  |
| **Laboratory parameters** |  |  |  |  |  |  |
| Calcium (mg/dL) | 0.02 |  | 0.18 | 0.17 |  | <0.001 |
| Phosphate (mg/dL) | 0.08 |  | <0.001 | 0.13 |  | <0.001 |
| PTH (pg/mL) | 0.07 |  | <0.001 | 0.03 |  | 0.06 |
| 25(OH) vitamin D (ng/mL) | -0.07 |  | <0.001 | -0.04 |  | 0.01 |
| eGFR (ml/min/1.73m^2^) | -0.21 |  | <0.001 | -0.20 |  | <0.001 |
| hs-CRP (mg/L) | 0.10 |  | <0.001 |  |  |  |
| Hemoglobin (g/dL) | -0.21 |  | <0.001 | -0.12 |  | <0.001 |
| MCV (fL) | -0.19 |  | <0.001 | -0.11 |  | <0.001 |
| Ferritin (µg/L)^*^ | -0.35 |  | <0.001 | -0.38 |  | <0.001 |
| TSAT (%)^*^ | -0.28 |  | <0.001 | -0.14 |  | <0.001 |
| sTfR (mg/L)^*^ | 0.46 |  | <0.001 | 0.27 |  | <0.001 |
| Hepcidin (ng/mL)^*^ | -0.31 |  | <0.001 | -0.31 |  | <0.001 |
| EPO (IU/L) | 0.32 |  | <0.001 | 0.21 |  | <0.001 |
| Glucose (mg/dL) | 0.07 |  | <0.001 | 0.04 |  | 0.009 |
| Smoking (% ) | 0.11 |  | <0.001 | 0.20 |  | <0.001 |
| Alcohol use (%) | -0.10 |  | <0.001 |  |  |  |
| NT-pro-BNP (pg/mL) | 0.14 |  | <0.001 | 0.05 |  | 0.001 |
| Total cholesterol (mg/dL) | -0.02 |  | 0.17 | 0.03 |  | 0.02 |
| ^*^Iron parameters have been placed separately in multivariate analyses, all reported coefficients of the other variables are from the multivariate model including ferritin. | | | | | | |
